# Managing egg allergy: A systematic review of traditional allergen avoidance methods and emerging graded exposure strategies

**DOI:** 10.1111/pai.70075

**Published:** 2025-04-01

**Authors:** Aoife Gallagher, Pamen Delgado Mainar, Caoimhe Cronin, Cristina Muñoz, Jesús Rodrigues Calleja, Conor Loughnane, Juan Trujillo

**Affiliations:** ^1^ Department of Paediatrics and Child Health University College Cork Cork Ireland; ^2^ Department of Paediatrics Cork University Hospital Cork Ireland; ^3^ Irish Centre for Maternal and Child Health Research (INFANT), HRB Clinical Research Facility Cork (CRF‐C) Cork University Hospital Cork Ireland; ^4^ Hospital Santa Ana Motril Spain; ^5^ Hospital Universitario Severo Ochoa Madrid Spain; ^6^ Hospital Rio Carrión Palencia Spain; ^7^ Cork University Business School University College Cork Cork Ireland

**Keywords:** allergen avoidance, dietary advancement therapy, egg allergy management, egg ladder, food allergy

## Abstract

Egg allergy represents a significant and growing health concern, particularly among young children. Consequently, there has been a surge in the development of management strategies to address this issue. While oral immunotherapy presents a promising novel approach, its resource‐intensive nature renders it impractical in many countries. This review aims to contrast the traditional method of strict avoidance with emerging, cost‐effective alternatives for managing egg allergy at home, such as the gradual introduction via a ladder approach. Studies were identified through the search of medical databases and gray literature, with a focus on studies spanning from 2003 to 2023. Studies were independently screened and appraised by two independent reviewers. One hundred and thirty‐four articles were identified. After removing duplicates and screening, 49 underwent full‐text review, resulting in 28 included articles. These encompassed various study designs and originated from multiple countries, primarily the USA, Australia and Canada. The interventions mainly focused on managing IgE‐mediated egg allergy through graded exposure to denatured/baked egg (*n* = 20), with an additional six studies exploring allergen avoidance and two studies investigating both management methods. A key observation from this review is the shift in management strategies towards incorporating methods such as graded exposure to denatured/baked egg alongside traditional allergen avoidance methods. Allergen avoidance remains the cornerstone of egg allergy management. However, there is a need for complementary approaches to optimise outcomes for individuals with egg allergy. Factors such as quality of life, including social inclusion and dietary diversity, as well as economic implications are crucial considerations.

**Abbreviations**
BMJ: British Medical Journal
BSACI: British Society for Allergy and Clinical Immunology
ESRC: Economic and Social Research Council
IgE: immunoglobulin E
MMAT: mixed methods appraisal tool
OFC: oral food challenge
PRISMA: preferred reporting items for systematic reviews and meta‐analyses
RCT: randomised controlled trial
SGEP: structured graduated exposure protocol
SPT: skin prick test
SpIgE: specific Immunoglobulin E

**Key message**

A key observation from this review is the shift in management strategies towards incorporating methods such as graded exposure to denatured/baked egg alongside traditional allergen avoidance methods. Allergen avoidance remains the cornerstone of egg allergy management. However, there is a need for complementary approaches to optimise outcomes for individuals with egg allergy. Factors such as quality of life, social inclusion, dietary diversity and economic implications are crucial considerations.

## 1 INTRODUCTION

Egg allergy is one of the most common food allergies in the world and is on the rise in recent years.^1^ So too are the management strategies employed in the management of egg allergy. While allergen avoidance is still the most commonly employed strategy in managing food allergies, there have been significant advancements in the field of egg allergy treatment in recent years.

One such method is oral immunotherapy (OIT), which involves gradual oral administration of food protein with the aim of reaching sustained unresponsiveness.^2^ There has been significant growth in research in egg OIT in recent years; however, this approach still carries the risk of anaphylaxis and requires significant resources and costs.

Another emerging method of IgE‐mediated egg allergy management is graded exposure to baked egg, often using a ladder approach. This strategy involves introducing small amounts of baked egg into the child's diet and gradually increasing the dose over time, with the ultimate goal of incorporating egg into the diet. It is a safe and effective method that has been shown to be well‐tolerated by children with egg allergy.^3^ The distinction between this management strategy and OIT remains ambiguous, as emerging evidence suggests that the ladder approach may have therapeutic effects in terms of inducing earlier tolerance.^4^, ^5^, ^6^ However, the graded exposure to baked egg approach has many other benefits including being less resource intensive than OIT as it can be done at home, empowering parents to take an active role in their child's treatment, as well as reducing the burden of hospital appointments for families and reducing healthcare costs.

Despite these new management strategies, traditional allergen avoidance remains the foundation of food allergy management, including egg allergy. This complete egg avoidance approach can be challenging, given the ubiquitous nature of egg in many processed foods, but remains an essential part of managing egg allergy. Careful reading of food labels and identification of egg‐containing ingredients is necessary to prevent accidental exposure. Parents, caregivers and individuals with egg allergy need to be educated on how to avoid egg‐containing foods and how to identify hidden sources of egg. Allergen avoidance alone can be challenging and can have a significant impact on quality of life, especially for children.^7^, ^8^ Therefore, it is essential to explore alternative management strategies.

This systematic review aims to gather and analyse published literature on traditional allergen avoidance methods and compare them to emerging methods of graded exposure to denatured egg protein. It is hoped that this review will provide valuable insights into the efficacy and safety of these methods and help healthcare providers and families make informed decisions about the best approach to managing egg allergy.

## 2 METHODS

The protocol for this systematic review is registered on PROSPERO (CRD42024550903). Reporting adheres to the guidelines outlined in the 2020 edition of the Preferred Reporting Items for Systematic Reviews and Meta‐Analyses (PRISMA) statement.

### 2.1 Search strategy

On 4th September 2024, a systematic search of medical databases including; Embase (*n* = 35), ProQuest Central (*n* = 20), MEDLINE (*n* = 29), Research Gate (*n* = 8), PubMed (*n* = 14), SpringerLink (*n* = 3), Wiley Online Library (n = 2), Web of Science (*n* = 2), Cochrane Library (*n* = 2), Scopus (*n* = 5), BMJ Journals (*n* = 1), as well as gray literature (*n* = 7) and citation search (*n* = 4) was conducted. The search string focused on the terms ‘IgE‐mediated egg allergy’, ‘management’ and ‘paediatric’, and was tailored to each database. After submission and peer review two further articles (*n* = 2) were identified and included in the review (Appendix S1).

### 2.2 Eligibility criteria

This study employed the PICOS framework—Participants, Intervention, Comparison, Outcome and Study design—to define eligibility criteria. Specifically, the study included the paediatric population (<16 years old) with IgE‐mediated egg allergy, where interventions for managing such allergies included allergen avoidance or graded exposure to heat denatured/baked egg. To maintain the focus of the review, interventions defining their approach as oral immunotherapy and those focusing on allergy prevention were excluded. All primary research study designs were considered to ensure the comprehensive coverage of the research conducted in this area. Given the heterogeneity of study designs, comparison was not used to include or exclude studies; instead, all reported outcomes were considered (Table 1).

**TABLE 1:** Eligibility criteria with rationale in PICOS format.

PICOS	Include	Exclude	Rationale
Population	Studies focusing on paediatric patients (patients <16 years) with IgE‐mediated egg allergy.	Studies on older patients (patients >16 years) or those with non‐IgE mediated egg allergy Studies focussing on food allergy other than egg	Egg allergy is the second most common food allergy in children. The majority of children will outgrow their egg allergy by school age, thus limiting the focus of allergy management to a younger population.
Intervention	Studies focusing on management of IgE‐mediated egg allergy either by allergen avoidance or by graded exposure to heat denatured/baked egg will be included.	Studies with oral immunotherapy (OIT) intervention or those focusing solely on pathogenesis, diagnosis, prevalence or allergy prevention will be excluded.	Although (OIT) presents a novel and promising approach to managing food allergies, its implementation can pose significant financial and resource burdens, rendering it unfeasible in many healthcare settings. This review aims to analyse the gold standard allergen avoidance with new, more cost‐effective methods of food allergy management such as home based introduction.
Comparison	Comparison will not be used as an inclusion criterion.	No exclusion on the basis of comparison.	As the studies included are not solely RCTs, there may be any or no comparison.
Outcome	Outcomes will not be used as an inclusion criterion.	No exclusion on the basis of outcomes.	All reported outcomes will be considered including; sustained unresponsiveness, successful reintroduction of egg to the diet, ongoing IgE‐mediated egg allergy and safety outcomes such as anaphylaxis.
Study design	Any type of primary research study design will be included; quantitative, qualitative or mixed method. Full text must be available.	Abstracts only, conference proceedings and opinion pieces will be excluded. Studies not published in English will be excluded	All primary research study designs will be considered to capture the full range of research conducted in this area.

### 2.3 Study selection

All citations identified were exported to Covidence software, which was utilised throughout the entire review process. Duplicates were removed. Two reviewers, AG and CC, independently evaluated articles based on their title and abstract, followed by a full‐text evaluation. In case of any disagreement, JT was designated as the third party to help resolve consensus issues. However, no disagreements persisted.

### 2.4 Quality appraisal

The methodological quality of each study was evaluated using the Mixed Methods Appraisal Tool (MMAT).^9^ This tool allows the appraisal of five categories of empirical studies: qualitative research, randomised controlled trials, non‐randomised studies, quantitative descriptive studies and mixed methods studies. AG completed the quality appraisal, and a random 33% was cross‐checked by CC. Findings were not used to exclude studies that met the inclusion criteria, as recommended in the tool's user guide. The MMAT is registered under copyright 1148552(10), and it was last updated in 2018.

### 2.5 Data extraction

Data was obtained by AG using Covidence software and a structured data extraction template. The data extracted included essential information such as the study's title, the authors, year of publication, the country in which the study was conducted, the method used to manage IgE‐mediated egg allergy, method of diagnosis of IgE‐mediated egg allergy, aim of the study, study design, population description, inclusion and exclusion criteria, total number of participants, main findings, strengths and weaknesses. To ensure the quality of the extracted data, 33% of the data was randomly cross‐checked by CC.

### 2.6 Data synthesis

The research included a wide range of study designs and produced both qualitative and quantitative findings. As per the guidance from the ESRC Methods Programme,^10^ descriptive summary tables were produced, and a narrative synthesis of the data was undertaken. The process involved four main elements: developing a theory of how and why the intervention works, developing a preliminary synthesis of findings, exploring relationships in the data and assessing the robustness of the synthesis. The content of the interventions was categorised into three groups: allergen avoidance (*n* = 6), graded exposure to heat denatured/baked egg (*n* = 20) or studies looking at both interventions (*n* = 2). Due to heterogeneity, meta‐analysis could not be undertaken.

## 3 RESULTS

### 3.1 Description of studies

**FIGURE 1:**
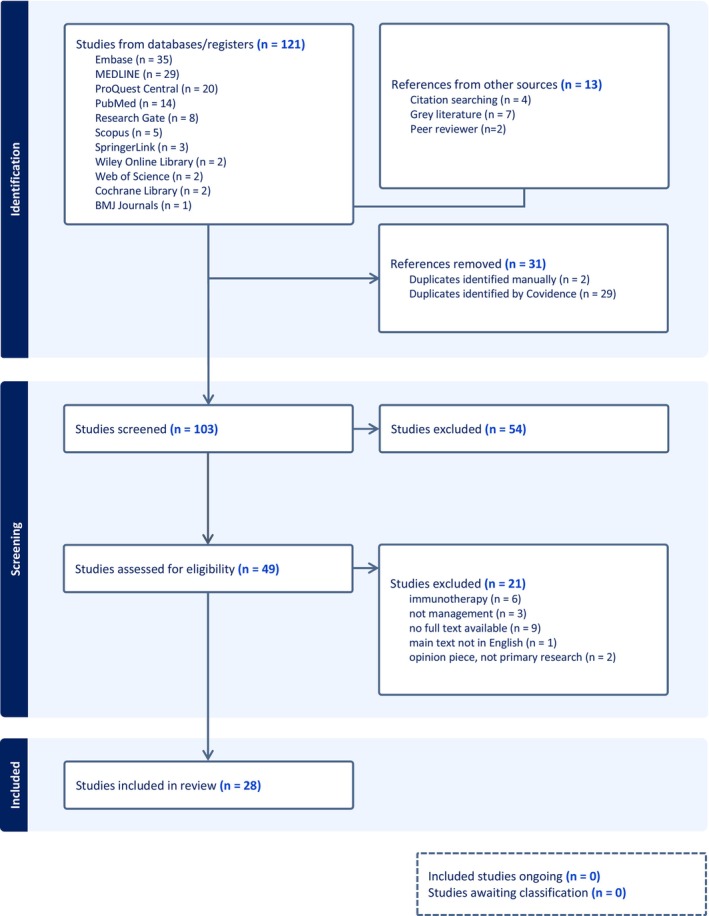
PRISMA flowchart.

A total of 132 articles were initially identified, with two subsequent articles identified by peer review. Following the removal of duplicates and screening by title and abstract, 49 articles were retained for full‐text screening. Ultimately, 28 articles were included (Figure 1). The included studies comprised various designs: review articles (*n* = 7), debates (*n* = 2), case–control study (*n* = 1), guidelines (*n* = 2), retrospective non‐randomised studies (*n* = 8), prospective non‐randomised studies (*n* = 2), cross‐sectional study (*n* = 2), systematic review (*n* = 2) and randomised control trials (*n* = 2). Interventions primarily originated from the USA (*n* = 8), Australia (*n* = 4) and Canada (*n* = 6), with three studies from Japan, two from Ireland and one each from Belgium, Greece, Israel, Poland and the UK. The studies generally featured small sample sizes, ranging from 26 to 243 participants, except for three outliers with 522, 458 and 881 participants. The predominant focus of interventions was on managing IgE‐mediated egg allergy through graded exposure to denatured/baked egg (*n* = 20), with an additional six studies exploring allergen avoidance and two studies investigating both management methods. Most studies used a combination of skin prick test (SPT), specific immunoglobulin E (SpIgE), oral food challenge (OFC) and clinical symptoms in the diagnosis of IgE‐mediated egg allergy, with one study using SPT alone, one using OFC alone and two using SpIgE alone (Table 2).

**TABLE 2:** Description of studies.

Study	Country	Method of Management	Diagnosis	Study Design	Population description	Total number of participants
Allen 2009	Australia	Avoidance	SPT; Clinical symptoms; OFC	Prospective study; Cross sectional study	Parents of children seen in a tertiary paediatric allergy clinic in 2003 and diagnosed with egg allergy	167
Anagnostou 2021	United States	Avoidance; Graded exposure to heat denatured/baked egg; OIT	SPT; SpIgE; Clinical symptoms; OFC	Review Article		
Bell 2008	United States	Avoidance	SpIgE; Clinical symptoms	Retrospective study	Patients with egg allergy seen in a tertiary referral clinic (Johns Hopkins Pediatric Allergy Clinic)	881
Caubet 2011	United States	Avoidance; OIT	SPT; SpIgE; Clinical symptoms; OFC	Review Article		
Chomyn 2022	Canada	Graded exposure to heat denatured/baked egg	SpIgE	Review Article		
Chomyn 2023	Canada	Graded exposure to heat denatured/baked egg				
Chua 2022	Canada			Review Article		
Clark 2010	United States	Avoidance	SPT; SpIgE; Clinical symptoms	Guideline		
Cotter 2021	Ireland	Graded exposure to heat denatured/baked egg	SPT; SpIgE; Clinical symptoms	Retrospective study	Retrospective chart review of data was collected from the paediatric allergy clinic, in Cork University Hospital.	29
Dang 2016	Australia	Avoidance	SPT; SpIgE; Clinical symptoms; OFC	Review Article; Debate	Paediatric patients with IgE‐mediated egg and milk allergy	
De Vlieger 2022	Belgium	Graded exposure to heat denatured/baked egg	SPT; SpIgE; Clinical symptoms; OFC	Randomised Control Trial	Baked egg tolerant children above 12 months of age	78
Gallagher 2024	Ireland	Graded exposure to heat denatured/baked egg	SPT; SpIgE; Clinical symptoms	Retrospective study	Children younger than 3 years diagnosed with IgE‐mediated milk and/or egg allergy between 2011 to 2021	458
Gotesdyner 2019	Israel	Graded exposure to heat denatured/baked egg	SPT; SpIgE; Clinical symptoms; OFC	Case control study	Infants <2 years with IgE‐mediated egg allergy	119
Heine 2006	Australia	Avoidance	SPT; SpIgE; Clinical symptoms; OFC	Review Article		
Johnston 2022	UK	Avoidance; Graded exposure to heat denatured/baked egg; OIT	SPT; SpIgE; Clinical symptoms	Guideline		
Kitamura 2021	Japan	Graded exposure to heat denatured/baked egg	SpIgE	Randomised controlled trial	Children aged 1–4 years	55
Konstantinou 2008	Greece	Graded exposure to heat denatured/baked egg	SPT; SpIgE; Clinical symptoms; OFC	Retrospective study	Patients referred to the food allergy unit of the department with a diagnosis of hen's egg allergy or sensitisation	94
Kotwal 2023	United states	Graded exposure to heat denatured/baked egg	SPT; SpIgE; Clinical symptoms; OFC	Retrospective study	Baked egg (BE) oral food challenges (OFC) that occurred between 2012 and 2019 at Johns Hopkins Pediatric Allergy Clinic	243
Krogulska 2011	Poland	Graded exposure to heat denatured/baked egg	OFC	Prospective study	Children diagnosed at the Department of Paediatric Allergology, Gastroenterology and Nutrition of the Medical University of Lodz and in the Clinic of Allergology at the Maria Konopnicka Memorial University Teaching Hospital No. 4 in Lodz because of the suspicion of allergy to cow's milk protein and/or egg white in the period from January 2005 to November 2009.	26
Leonard 2016	United States	Graded exposure to heat denatured/baked egg	SPT; SpIgE; OFC	Review Article; Debate	Paediatric patients with IgE‐mediated egg or milk allergy	
Mack 2023	Canada	Graded exposure to heat denatured/baked egg		Review Article	Children diagnosed with either IgE‐mediated or non‐IgE‐mediated milk or egg allergies.	
Okada 2016	Japan	Graded exposure to heat denatured/baked egg	SpIgE; Clinical symptoms; OFC	Non‐randomised experimental study; Retrospective study	Paediatric patients with IgE‐mediated egg allergy	197
Thomas 2021	Australia	Graded exposure to heat denatured/baked egg	SPT	Non‐randomised experimental study; Retrospective study	Patients with mild to moderate IgE‐mediated egg allergy aged <18 years.	47
Upton 2018	Canada	Graded exposure to heat denatured/baked egg	SPT; SpIgE	Review Article		
Upton 2023	United States	Avoidance; Graded exposure to heat denatured/baked egg	SPT; SpIgE; Clinical symptoms; OFC	Cross sectional study	North American Academy of Allergy, Asthma & Immunology members offering BM and BE introduction in 2021.	72
Upton 2024	Canada	Graded exposure to heat denatured/baked egg		Review Article		
Venter 2022	United States	Graded exposure to heat denatured/baked egg		Systematic review	Currently published food allergen ladders	
Yamashita 2024	Japan	Graded exposure to heat denatured/baked egg	SpIgE; Clinical symptoms; OFC	Retrospective study; Case series	Patients diagnosed with IgE‐related HE allergy who underwent the HE OFC at Showa University Hospital between January 1, 2012 and March 31, 2023	522

### 3.2 Interventions and outcomes

#### 3.2.1 Allergen avoidance

Eight studies have examined the effectiveness of allergen avoidance for managing IgE‐mediated egg allergy. As the traditional method of food allergy management, allergen avoidance remains the standard of care in many countries. Six studies focused solely on allergen avoidance, while two explored a combination of both allergen avoidance as the cornerstone of egg allergy management but also with a role for baked egg introduction.^11^, ^12^

All eight studies agree that egg allergy is common and generally has a good prognosis. One study reported that although egg allergy has a good prognosis, with most children naturally acquiring tolerance, this process may not occur as young as previously thought and is consistently longer in those with atopic disease.^13^ Another study found that despite stricter advice regarding allergen avoidance and fewer accidental exposures and reactions, rates of tolerance acquisition or time to achieve tolerance were unaffected.^14^

Most studies agree that although allergen avoidance remains the cornerstone of management, there is a role for new emerging strategies such as OIT^15^ or the introduction of baked egg to the diet.^16^ Four studies mentioned the role of baked egg in egg allergy management, all agreeing that the introduction of baked egg to the diet can be achieved, especially in those with milder allergy.^17^

The 2021 guideline from the British Society for Allergy and Clinical Immunology (BSACI)^12^ recognises that while allergen avoidance is still the primary management approach, prolonged exclusion of egg from the diet can lead to persistent allergy and increase dietary and social exclusion.

Two studies also explored the need for a maternal exclusion diet when breastfeeding.^12^, ^18^ Both found that there is no evidence for a protective effect of a maternal elimination diet during pregnancy and that most infants with egg allergy should continue to breastfeed, with their mother on an unrestricted diet. However, both studies also agreed that there may be a potential need for maternal egg exclusion in babies with persistent atopic dermatitis with egg as a trigger.

#### 3.2.2 Graded exposure to denatured/baked egg

Graded exposure to denatured/baked egg was the most common treatment method for children with IgE‐mediated egg allergy. Twenty‐two studies have examined this approach, but the term ‘graded exposure to denatured/baked egg’ can refer to different methods. Eight studies used the word ‘ladder’ to describe their approach,^3^, ^19^, ^20^, ^21^, ^22^, ^23^, ^24^, ^25^ two used a stepwise oral food challenge (OFC) protocol,^26^, ^27^ two used the term ‘dietary advancement therapy’,^23^, ^24^ another used a structured graduated exposure protocol (SGEP),^5^ with the remaining introducing various forms of heated or baked egg to the diet, with or without OFC. One study provided guidance on how to standardise the introduction of baked egg to the diet via a ladder approach.^21^ This involves considering the dose, timing, temperature, wheat matrix, cultural aspects and taste and texture of the foods included on the ladder. Another study also provided guidance in the form of a checklist for potentially suitable patients.^19^

Most studies report that introducing baked egg to the diet is safe and well tolerated, especially in milder cases. However, some studies report that this approach is also safe and effective in moderate and severe cases also.^3^, ^23^, ^27^, ^28^ Overall rates of baked egg tolerance in egg allergy children are as high as 70%–92.7%.

Although well tolerated, this approach is not without risk. Three studies specifically mentioned the need for careful consideration of the type of patient suitable,^4^, ^19^, ^25^ with four more reporting the need for OFC to confirm baked egg tolerance before introducing baked egg at home.^28^, ^29^, ^30^, ^31^

While the ladder approach is not without risk, it has several advantages. Ten studies mentioned the increase in tolerance acquisition rates,^4^, ^5^, ^6^, ^22^, ^25^, ^28^, ^29^, ^30^, ^32^, ^33^ a further seven mentioned the advantages in widening the diet of patients and their families, improving both nutrition as well as six studies mentioning the improvement in quality of life and social inclusion.^5^, ^6^, ^22^, ^29^, ^30^, ^31^, ^34^

Some studies suggest that the ladder approach is an active form of treatment, with immunological changes occurring as patients progress along the ladder. However, immunological markers such as skin prick testing (SPT) and egg specific IgE (SpIgE) do not predict the acquisition of tolerance^30^ or initial reaction severity.^4^, ^6^ One study suggests that classifying patients into ‘heated egg yolk slightly contaminated with egg white reactive’ or ‘heated egg yolk slightly contaminated with egg white tolerant’ may be useful, but further research is needed in this area.^35^ Similarly, another study found that absolute levels of egg‐specific serum IgE cannot predict the outcome of baked egg OFC.^4^ The inability to predict reactions and/or tolerance is the reason why some studies recommend the ladder approach for all patients with IgE‐mediated egg allergy regardless of severity.

Three studies mention the advantages of the ladder approach in a resource‐scarce setting.^3^, ^6^, ^20^, ^21^ Two specifically recommended the appropriateness of the ladders for virtual or telehealth consultations, acknowledging their effectiveness in reducing the strain on limited resources.

### 3.3 Quality appraisal

All 28 studies satisfactorily met the screening criteria of the MMAT. Although seven studies were marked as ‘can't tell’, it was mainly due to reporting rather than methodological limitations, specifically concerning confounders and risk of non‐response bias. One study was a quantitative randomised controlled trial which utilised open food challenges and involved parents giving their child doses of boiled egg at home.^26^ As a result, it was not double‐blinded and received a ‘no’ for that criterion. Similarly, another randomised control trial does not specifically state if assessors were blinded; however, since the study was conducted at home with parents managing the reintroduction, blinding them to the protocol group may have been difficult. Although it would be possible to blind assessors to the groups the study does not specifically state this and so the study was given ‘can't tell’ for that criterion.^30^ Two studies were deemed to have incomplete data. In one quantitative non‐randomised study only 76% of participants received the questionnaire, with 84% of these responding.^14^ Another was a randomised control trial in which 90% participated in the intention to treat analysis, with only 67% of the original cohort completing the protocol.^30^ The acceptable complete data value from previous studies ranged from 80%^36^, ^37^ to 95%.^38^ Eighteen studies were identified as having no methodological quality issues and were marked as ‘yes’ in all categories. (Table 3).

**TABLE 3:** Quality appraisal of the included articles using the mixed methods appraisal tool.

Study	S1	S2	Study Design	C1	C2	C3	C4	C5	Comment
Allen 2009	Y	Y	Quantitative Non‐randomised study	Y	Y	N	CT	Y	76% participants received the questionnaire, with 84% of these responding. From previous studies acceptable complete data value ranged from 80% (Thomas et al., 2004; Zaza et al., 2000) to 95% (Higgins et al., 2016). Fisher exact test was used to determine if there are non‐random associations between two categorical variables; however, it is unclear whether the researchers used any regression models to account for potential confounders.
Anagnostou 2021	Y	Y	Qualitative Study	Y	Y	Y	Y	Y	
Bell 2008	Y	Y	Quantitative Non‐randomised study	Y	Y	Y	CT	Y	While the text discusses the methods used to analyse the development of egg tolerance over time and compares different groups based on clinical characteristics and IgE levels, it doesn't explicitly mention whether confounders were controlled for in the analysis.
Caubet 2011	Y	Y	Qualitative Study	Y	Y	Y	Y	Y	
Chomyn 2022	Y	Y	Qualitative Study	Y	Y	Y	Y	Y	
Chomyn 2023	Y	Y	Quantitative Non‐randomised study	Y	Y	N	CT	Y	Only 53 of 109 participants responded to follow‐up surveys. The study does not explicitly mention adjusting for confounders such as the severity of the initial allergy, concurrent allergies or other atopic conditions (though atopic history was collected in the baseline survey). Without adjustments or stratified analysis, it is unclear if these factors could have influenced the outcomes.
Chua 2022	Y	Y	Qualitative Study	Y	Y	Y	Y	Y	
Clark 2010	Y	Y	Qualitative Study	Y	Y	Y	Y	Y	
Cotter 2021	Y	Y	Quantitative descriptive study	Y	Y	Y	CT	Y	The study does not provide information on the risk of nonresponse bias, as it did not mention the response rate of the participants.
Dang 2016	Y	Y	Qualitative Study	Y	Y	Y	Y	Y	
De Vlieger 2022	Y	Y	Quantitative randomised controlled trial	Y	Y	N	CT	Y	90% were included in the intention‐to‐treat analysis. 67% of the initial cohort completed the full protocol. It is not specifically reported if outcome assessors were blinded
Gallagher 2024	Y	Y	Quantitative descriptive study	Y	Y	Y	Y	Y	
Gotesdyner 2019	Y	Y	Quantitative Non‐randomised study	Y	Y	Y	Y	Y	
Heine 2006	Y	Y	Qualitative Study	Y	Y	Y	Y	Y	
Johnston 2022	Y	Y	Qualitative Study	Y	Y	Y	Y	Y	
Kitamura 2021	Y	Y	Quantitative randomised controlled trial	Y	Y	Y	N	Y	This study used open food challenges and involved parents preparing and administering doses of boiled egg to their child at home and so was not double‐blinded.
Konstantinou 2008	Y	Y	Quantitative Non‐randomised study	Y	Y	Y	CT	Y	The article does not explicitly mention any confounders that were accounted for in the design and analysis.
Kotwal 2023	Y	Y	Quantitative Non‐randomised study	Y	Y	Y	Y	Y	
Krogulska 2011	Y	Y	Quantitative Non‐randomised study	Y	Y	Y	CT	Y	Although researchers followed recommended EAACI guidelines and used exclusion criteria, the study does not provide detailed information about the confounders that were accounted for in the design and analysis of the study.
Leonard 2016	Y	Y	Qualitative Study	Y	Y	Y	Y	Y	
Mack 2023	Y	Y	Quantitative Non‐randomised study	Y	Y	Y	Y	Y	
Okada 2016	Y	Y	Quantitative Non‐randomised study	Y	Y	Y	Y	Y	
Thomas 2021	Y	Y	Mixed methods study	Y	Y	Y	Y	Y	
Upton 2018	Y	Y	Qualitative Study	Y	Y	Y	Y	Y	
Upton 2023	Y	Y	Quantitative descriptive study	Y	CT	Y	N	Y	The sample consists of a randomly generated selection of AAAAI members, which increases the likelihood of representativeness. However, the low response rate of 10.1% might limit how well the sample reflects the broader target population. While response attrition is common in electronic surveys, there may be nonresponse bias, where those who participated could differ from those who did not in terms of their practices or experiences. With a response rate of only 10.1%, there is a moderate‐to‐high risk of nonresponse bias. Those who responded may have different views or practices compared to non‐respondents, particularly since surveys related to clinical practices can often attract those with strong opinions or established protocols on BM/BE introduction.
Venter 2022	Y	Y	Qualitative Study	Y	Y	Y	Y	Y	
Yamashita 2024	Y	Y	Quantitative Non‐randomised study	Y	Y	Y	Y	Y	
Upton 2024	Y	Y	Qualitative Study	Y	Y	Y	Y	Y	

*Note*: See the MMAT tool for full description of each category criteria. Comments are given for categories that are marked ‘no’ or ‘can't tell’.

Abbreviations: C, criterion of different study design; CT, can't tell; N, no; S, screening question; Y, yes.

## 4 DISCUSSION

Egg allergy represents a significant and growing health concern, particularly among young children. As highlighted in this systematic review, managing egg allergy presents a multifaceted challenge requiring an understanding of traditional allergen avoidance methods as well as emerging graded exposure strategies such as the egg ladder. The findings of this review offer valuable insights for healthcare providers and families of children with IgE‐mediated egg allergy.

One of the key observations from this review is the shift in management strategies towards incorporating methods such as graded exposure to denatured/baked egg alongside traditional allergen avoidance methods. Allergen avoidance remains the cornerstone of egg allergy management. However, despite stringent adherence to allergen avoidance measures, rates of tolerance acquisition or time to achieve tolerance may remain unaffected, as evidenced by the findings of Allen et al. This emphasises the need for complementary approaches to optimise outcomes for individuals with egg allergy.

Graded exposure to denatured/baked egg has emerged as a promising strategy in the management of egg allergy, with several studies reporting its safety and efficacy, particularly in milder cases. This review assesses diverse methodologies employed in graded exposure protocols, ranging from ladder approaches to structured oral food challenge (OFC) protocols. These strategies offer a gradual introduction of baked egg, aiming to ultimately facilitate the incorporation of egg freely into the diet. The high rates of baked egg tolerance reported in the literature underscore the potential of this approach to broaden dietary options and improve quality of life for individuals with egg allergy. Many studies also highlight the safety of baked/cooked egg introduction, with one study by Yanagida et al. demonstrating high rates of tolerating low and medium dose cooked egg with very few severe reactions and no patients requiring intramuscular adrenaline^39^ Many studies also recognise that gradual exposure to baked egg is an active treatment and potential form of immunotherapy promoting earlier tolerance and allergy resolution than allergen avoidance alone.

However, the review also highlights several considerations and challenges associated with graded exposure strategies. While generally well‐tolerated, the risk of adverse reactions necessitates careful patient selection and monitoring, as emphasised by the need for OFC confirmation of baked egg tolerance in some cases. Furthermore, the heterogeneity in study methodologies and outcome measures complicates direct comparisons and limits the generalisability of findings. As highlighted by Venter et al., Mack et al. and Chua et al., there is a need for standardisation of ladder protocols as well as validated checklists for patient suitability and safety. Future research efforts should aim to standardise protocols and adopt consistent outcome measures to facilitate more robust comparisons across studies. This critical need for methodological rigor and standardisation has also been addressed in the recent meta‐analysis by Anagnostou et al. (2024),^40^ which provided valuable insights into the current limitations of dietary advancement therapy research. Their meticulous analysis revealed that despite promising trends in desensitisation outcomes, the field would benefit substantially from addressing heterogeneity between studies, implementing consistent outcome reporting measures, and standardising study products, duration, populations and assessment methods—ultimately advancing the quality of evidence supporting graded exposure approaches in egg allergy management.

This review also underscores the importance of considering broader implications beyond clinical efficacy when evaluating management strategies for egg allergy. Quality of life considerations, such as the impact of allergen avoidance on social inclusion and dietary diversity, warrant consideration in decision‐making processes. Additionally, the review highlights the potential economic implications associated with different management approaches, with graded exposure methods offering potential cost‐saving benefits compared to resource‐intensive interventions like oral immunotherapy.

## 5 STRENGTHS AND LIMITATIONS

### 5.1 Strengths

This review follows the Preferred Reporting Items for Systematic Reviews and Meta‐Analyses (PRISMA) guidelines, which are widely recognised standards for conducting systematic reviews. The utilisation of the PICOS framework for defining eligibility criteria ensures clarity and consistency in study selection. The review encompasses a wide range of studies with various study designs such as review articles, debates, case–control studies, guidelines and both retrospective and prospective non‐randomised studies. Employing a narrative synthesis approach to analyse the data is appropriate given the heterogeneity of the included studies. Descriptive summary tables organise the findings, facilitating clear presentation and interpretation of the results. The methodological quality of each study is assessed using the Mixed Methods Appraisal Tool (MMAT), allowing for a critical evaluation of the included studies. The review discusses the clinical implications of the findings, highlighting the potential benefits and challenges associated with each management strategy. This information is valuable for healthcare providers and families in making informed decisions about the management of IgE‐mediated egg allergy. Overall, the strengths of this review lie in its comprehensive coverage of the literature, rigorous methodology, transparent reporting and clear presentation of findings with clinical relevance.

### 5.2 Limitations

While this review demonstrates several strengths, it also has some limitations. Despite efforts to conduct a systematic search and employ rigorous methodology, there may still be inherent biases in selecting studies, such as publication bias and reliance on English‐language publications.

Despite efforts to include various study designs, the review only identified two RCTs related to managing egg allergy. RCTs are considered the gold standard for evaluating interventions, as they minimise bias and provide more substantial evidence for causal relationships. The limited availability of RCTs may affect the robustness of the conclusions drawn from the review. Many of the included studies also have small sample sizes, which may limit the statistical power and reliability of the findings.

Although the review covers traditional allergen avoidance methods as well as emerging graded exposure strategies, it excludes other potential interventions such as oral immunotherapy (OIT) and allergy prevention strategies. This narrow focus may limit the comprehensiveness of the review and overlook alternative approaches to managing egg allergy.

Many of the included studies did not have long‐term follow‐up data to assess the sustained efficacy and safety of the interventions. Long‐term outcomes, such as the development of sustained unresponsiveness or the recurrence of allergic reactions over time, are crucial for evaluating the effectiveness of allergy management strategies.

## 6 CONCLUSION

This systematic review on managing egg allergy provides valuable insights into traditional allergen avoidance methods and the promising benefits of emerging graded exposure strategies. While allergen avoidance remains the foundation of food allergy management, it can be challenging, given the ubiquitous nature of egg in many processed foods. On the other hand, emerging methods of graded exposure to baked egg have been shown to be safe and effective, and can be done at home, reducing the burden of hospital appointments for families and healthcare costs. However, it is important to note that graded exposure may not be suitable for all children and should be done under medical supervision.

Overall, this systematic review provides healthcare providers and families with valuable information to make informed decisions about the best approach to managing egg allergy. It highlights the need for individualised management strategies, taking into account the child's age, severity of allergy and family preferences. Future research should focus on refining and standardising protocols, as well as assessing their long‐term effectiveness and safety in all cohorts of patients with egg allergy.

## AUTHOR CONTRIBUTIONS

**Aoife Gallagher:** Conceptualization; methodology; data curation; funding acquisition; investigation; project administration; visualization; writing – original draft; writing – review and editing; formal analysis. **Pamen Delgado Mainar:** Conceptualization; investigation; resources; writing – review and editing. **Caoimhe Cronin:** Data curation; formal analysis; investigation; project administration; visualization; writing – original draft; writing – review and editing. **Cristina Muñoz:** Conceptualization; investigation; resources; writing – review and editing; validation. **Jesús Rodrigues Calleja:** Conceptualization; investigation; resources; writing – review and editing. **Conor Loughnane:** Software; investigation; writing – review and editing. **Juan Trujillo:** Conceptualization; methodology; funding acquisition; resources; supervision; writing – review and editing.

### PEER REVIEW

The peer review history for this article is available at https://www.webofscience.com/api/gateway/wos/peer‐review/10.1111/pai.70075.

## Supporting information


Appendix S1.
