# Supplementary material for: Managing egg allergy: A systematic review of traditional allergen avoidance methods and emerging graded exposure strategies
Source: Pediatr Allergy Immunol. 2025 Apr 1;36(4):e70075. doi: 10.1111/pai.70075 (PMC11960040; doi:10.1111/pai.70075)
Supplement: Supplementary file 1 — Appendix S1. [file PAI-36-e70075-s001.docx]

# Search strings

('egg allergy'/exp OR 'egg allergy') AND ('ige mediated':ti,ab,kw OR ige:ti,ab,kw OR 'immunoglobulin e':ti,ab,kw OR 'immediate type hypersensitivity':ti,ab,kw) AND (paediatric:ti,ab,kw OR pediatric:ti,ab,kw OR child:ti,ab,kw OR children:ti,ab,kw) AND (management:ti,ab,kw OR strategy:ti,ab,kw OR treatment:ti,ab,kw) NOT (prevention:ti,ab,kw OR prevent:ti,ab,kw OR predict:ti,ab,kw OR predicting:ti,ab,kw) NOT (vaccination:ti,ab,kw OR vaccine:ti,ab,kw OR immunisation:ti,ab,kw OR immunization:ti,ab,kw) NOT anaphylaxis:ti,ab,kw NOT 'oral immunotherapy':ti,ab,kw NOT (gord:ti,ab,kw OR gerd:ti,ab,kw OR 'gastroesophageal reflux':ti,ab,kw) AND egg:ti AND [2003-2023]/py

('egg allergy') AND ('ige mediated' OR ige OR 'immunoglobulin e' OR 'immediate type hypersensitivity') AND (paediatric OR pediatric OR child OR children) AND (management OR strategy OR treatment) NOT (prevention OR prevent OR predict OR predicting) NOT (vaccination OR vaccine OR immunisation OR immunization) NOT anaphylaxis NOT 'oral immunotherapy’ NOT (gord OR gerd OR 'gastroesophageal reflux') NOT (eosinophilic OR EOE)

Grey literature search: egg allergy paediatric “ige mediated egg allergy”, 2003-2023
